# Supplementary material for: l-Ornithine-N5-monooxygenase (PvdA) Substrate Analogue Inhibitors for Pseudomonas aeruginosa Infections Treatment: Drug Repurposing Computational Studies
Source: Biomolecules. 2022 Jun 25;12(7):887. doi: 10.3390/biom12070887 (PMC9313252; doi:10.3390/biom12070887)
Supplement: Supplementary file 1 [file biomolecules-12-00887-s001.zip › biomolecules-1770521-supplementary.pdf]

Rosy et al. L-ornithine-N5-monooxygenase (PvdA) substrate analogue inhibitors for *Pseudomonas aeruginosa* infections treatment: Drug repurposing computational studies

Supplementary Tables

Table S1. Binding energies of drugs similar to L-ornithine, docked with PvdA containing $NADP^+$ and FAD at pH 8.3

S. No	Name of the Drug	Binding Energy (kcal mol^{-1})
1.	2,6-Diaminopimelic Acid	−6
2.	2-Aminopimelic Acid	−5.9
3.	6-hydroxy-L-norleucine	−5.5
4.	(2s)-2,8-Diaminooctanoic Acid	−5.5
5.	2-Methylleucine	−5.5
6.	Allo-Isoleucine	−5.5
7.	2-Amino-6-Oxo-Hexanoic Acid	−5.4
8.	D-Glutamine	−5.4
9.	5-Hydroxy Norvaline	−5.3
10.	4-Carboxy-4-Aminobutanal	−5.3
11.	D-Lysine	−5.2
12.	D-Leucine	−5.1
13.	Delta-Amino Valeric Acid	−4.7
14.	Norvaline	−4.6
15.	Alpha-Aminobutyric Acid	−4.5

Table S2: Binding energies of drugs that has substructures like L-ornithine, docked with PvdA containing $NADP^+$ and FAD at pH 8.3

S. No	Name of the drug	Binding Energy (Kcal/mol)
1.	Argininosuccinate	−6.7
2.	Glutathione sulfinate	−6.7
3.	N-omega-nitro-L-arginine methyl ester	−6.6
4.	Glutathione	−6.4
5.	Nitroarginine	−6.4
6.	S-Methyl-Glutathione	−6.4
7.	N-Alpha-L-Acetyl-Arginine	−6.3
8.	N-Acetyl-L-Citrulline	−6.3
9.	N~2~-Succinylornithine	−6.3
10.	S-Hydroxymthyl Glutathione	−6.3
11.	Glutamine t-butyl ester	−6.1
12.	N,N-dimethylarginine	−6
13.	N-(Phosphonoacetyl)-L-Ornithine	−6
14.	L-Eflornithine	−6
15.	gamma-Glutamylcysteine	−6
16.	Glutamine hydroxamate	−5.9
17.	Aceglutamide	−5.9
18.	N3, N4-Dimethylarginine	−5.8
19.	5-N-Allyl-Arginine	−5.8
20.	Tilarginine	−5.8
21.	L-Citrulline	−5.7
22.	N5-Methylglutamine	−5.7
23.	L-Thiocitrulline	−5.5
24.	Cilazapril	−3.8
25.	Glutathione disulfide	−2.6
26.	S-P-Nitrobenzyloxycarbonylglutathione	−1.1
27.	Talotrexin	4.2
28.	CTT-1057	6.7
29.	Davunetide	34
30.	Trypanothione	54.8

Supplementary Figures

MD Simulations Run 2

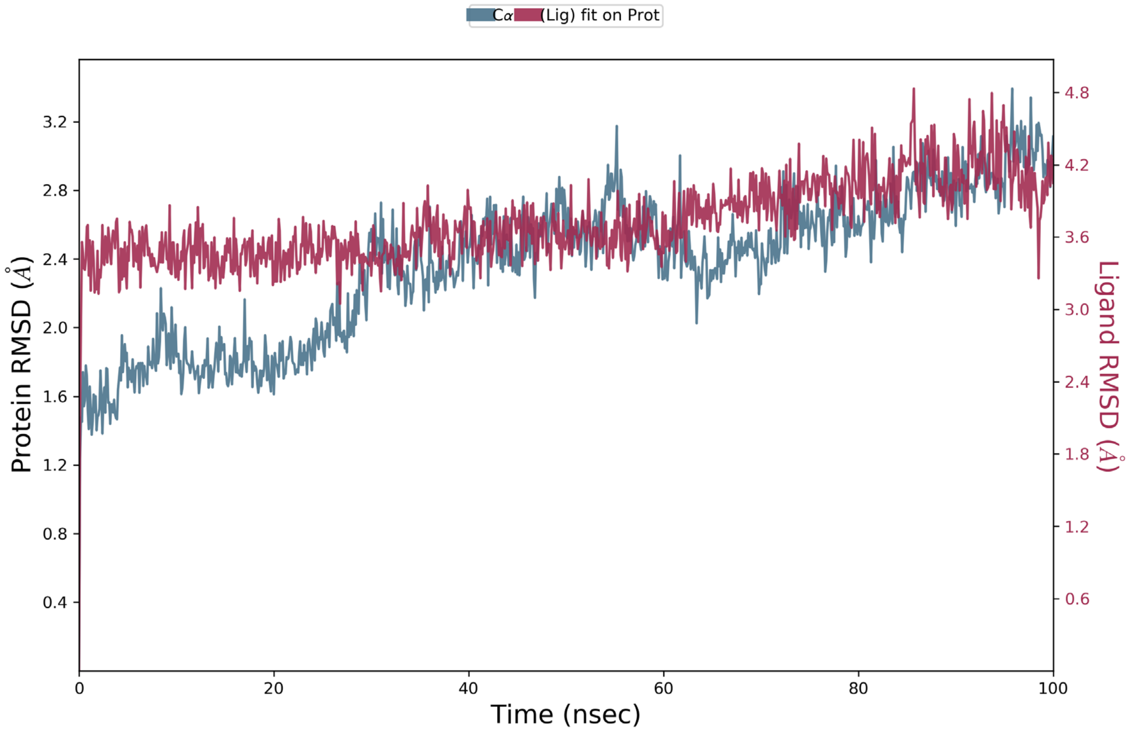

Figure S1: RMSD of protein and ligand throughout the 100 ns simulation; The interaction of PvdA and N-2-succinyl-ornithine were found to be stable after 40 ns.

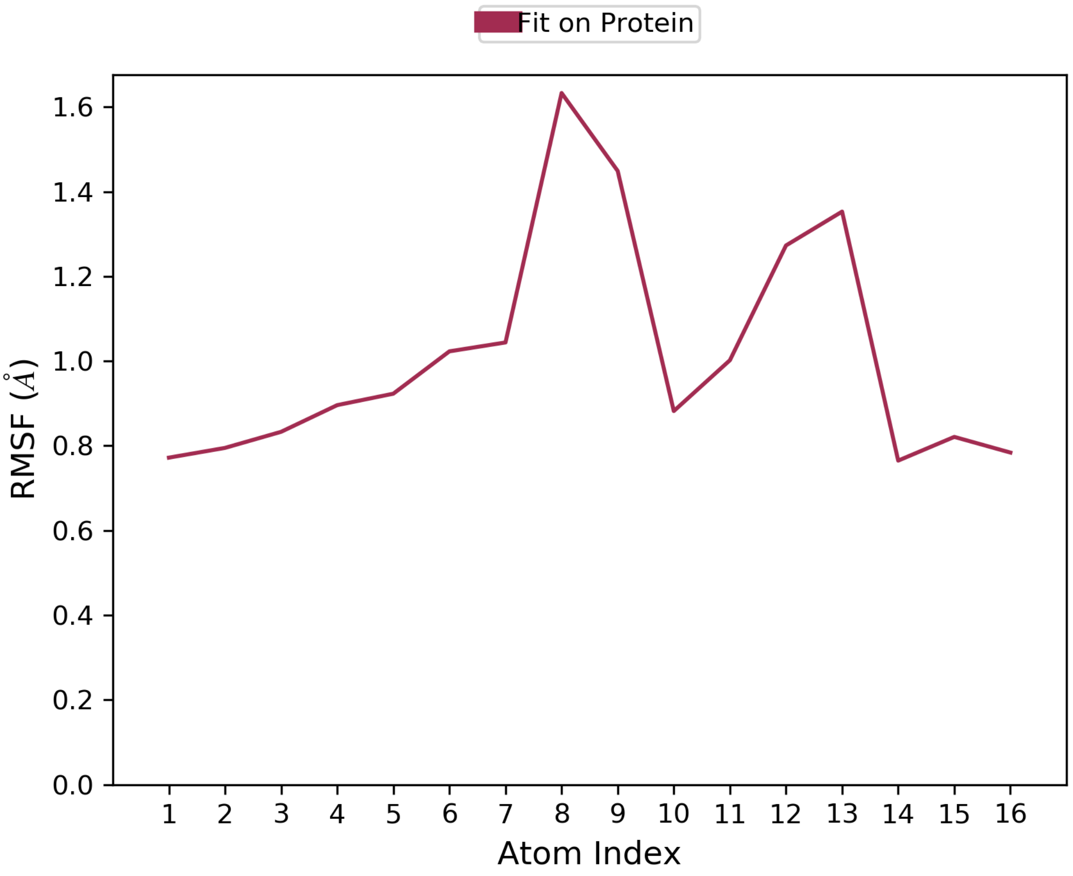

Figure S2: Ligand RMSF plot for characterizing changes in the ligand atom positions. The RMSF of all the atoms are not more than 2Å, which indicates that the binding is stable.

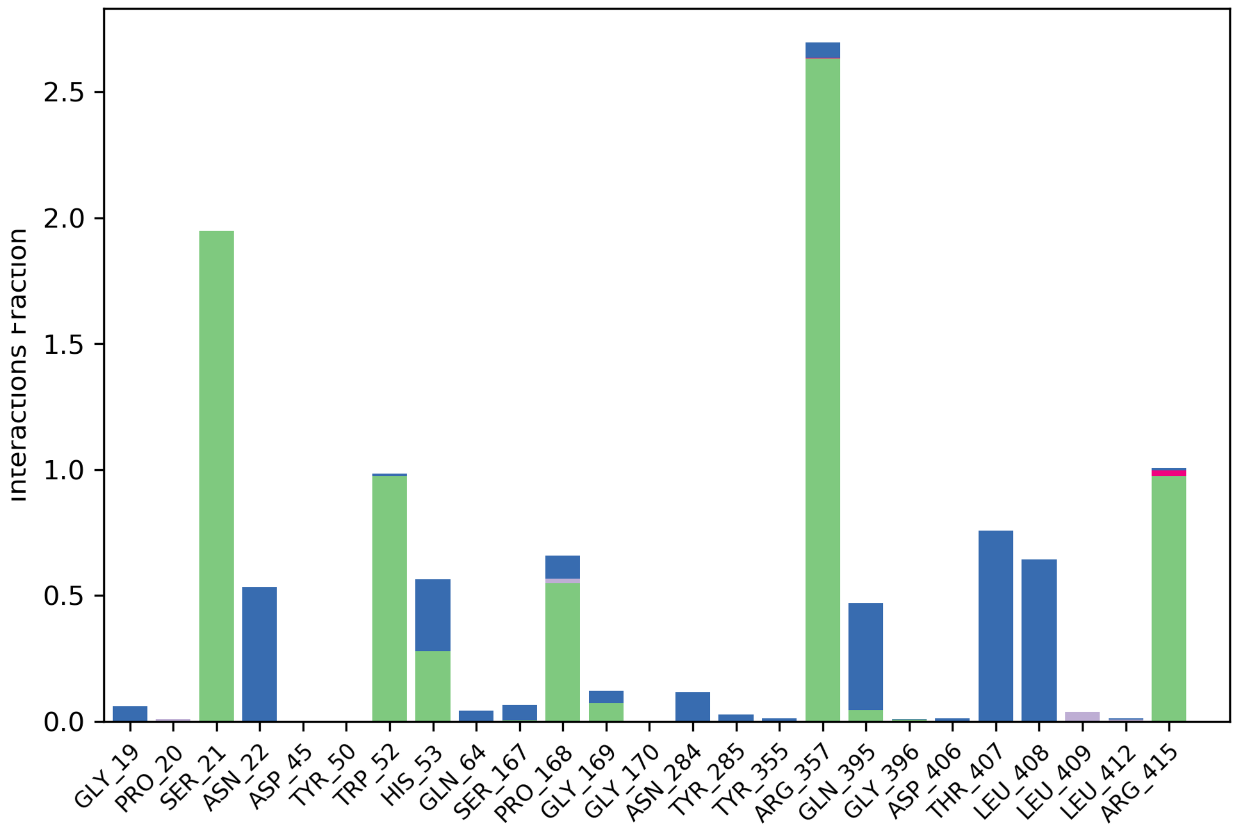

Figure S3: Various interactions of the ligand with the amino acids are represented. Amino acids of PvdA that make interactions with ligand and the type of interaction are provided. Green- Hydrogen Bond; Red-Ionic; Lavender-Hydrophobic; Blue-Water Bridge. Arg357 is found to make three kinds of interactions, namely, a hydrogen bond, water-bridge and ionic interaction.

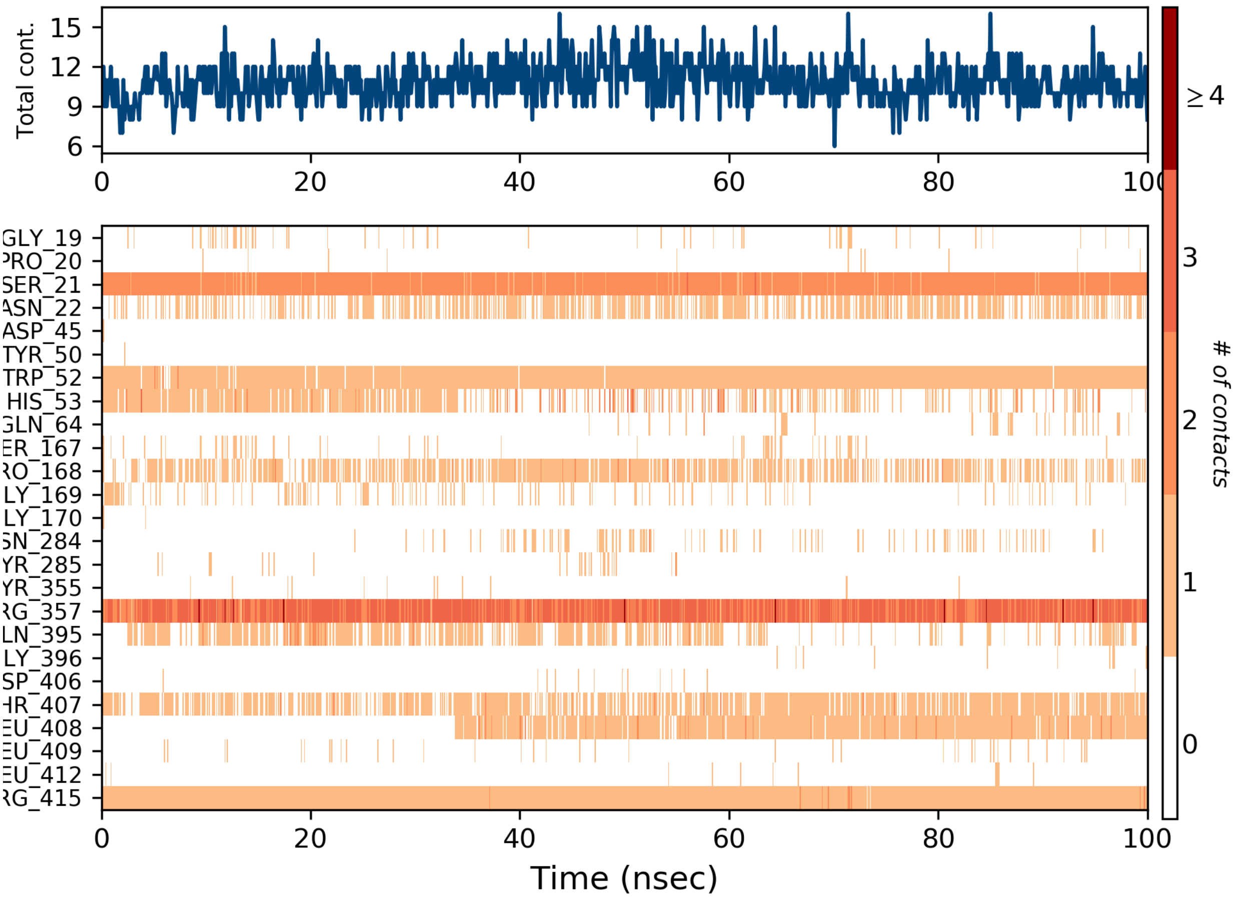

Figure S4: The number of contacts made by the ligand throughout the 100 ns simulation is shown in the top panel. Some residues make more than one specific contact with the ligand, which is represented by a darker shade of orange in the bottom panel, according to the scale to the right of the plot. Arg357 was found to make more than one single contact with the ligand.

MD Simulations Run 3:

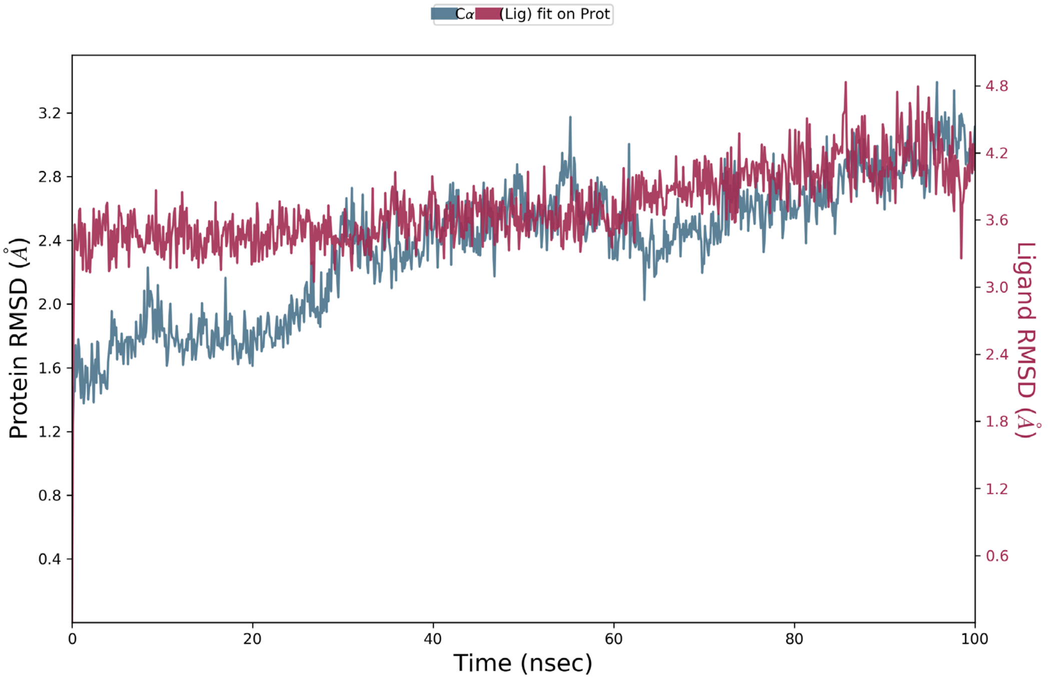

Figure S5: RMSD of protein and ligand throughout the 100 ns simulation; The interaction of PvdA and N-2-succinyl-ornithine were found to be stable after 40 ns.

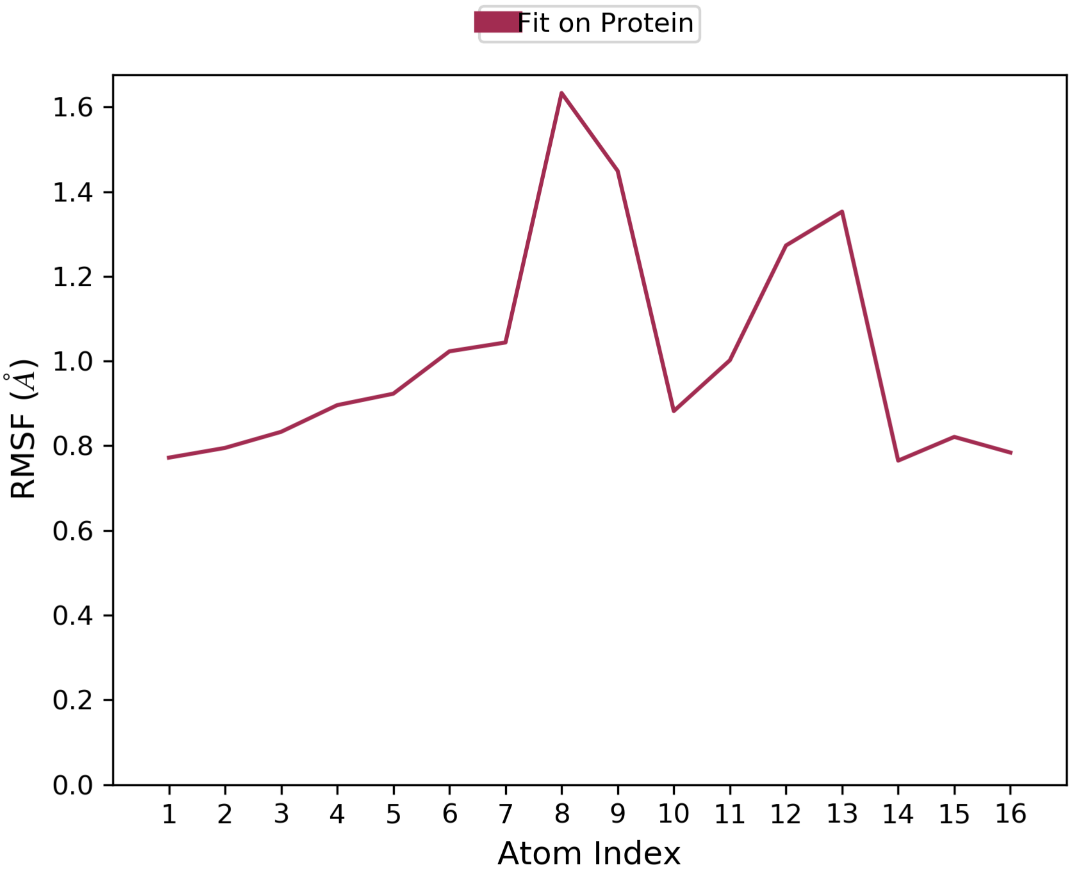

Figure S6: Ligand RMSF plot for characterizing changes in the ligand atom positions. The RMSF of all the atoms are not more than 2Å, which indicates that the binding is stable.

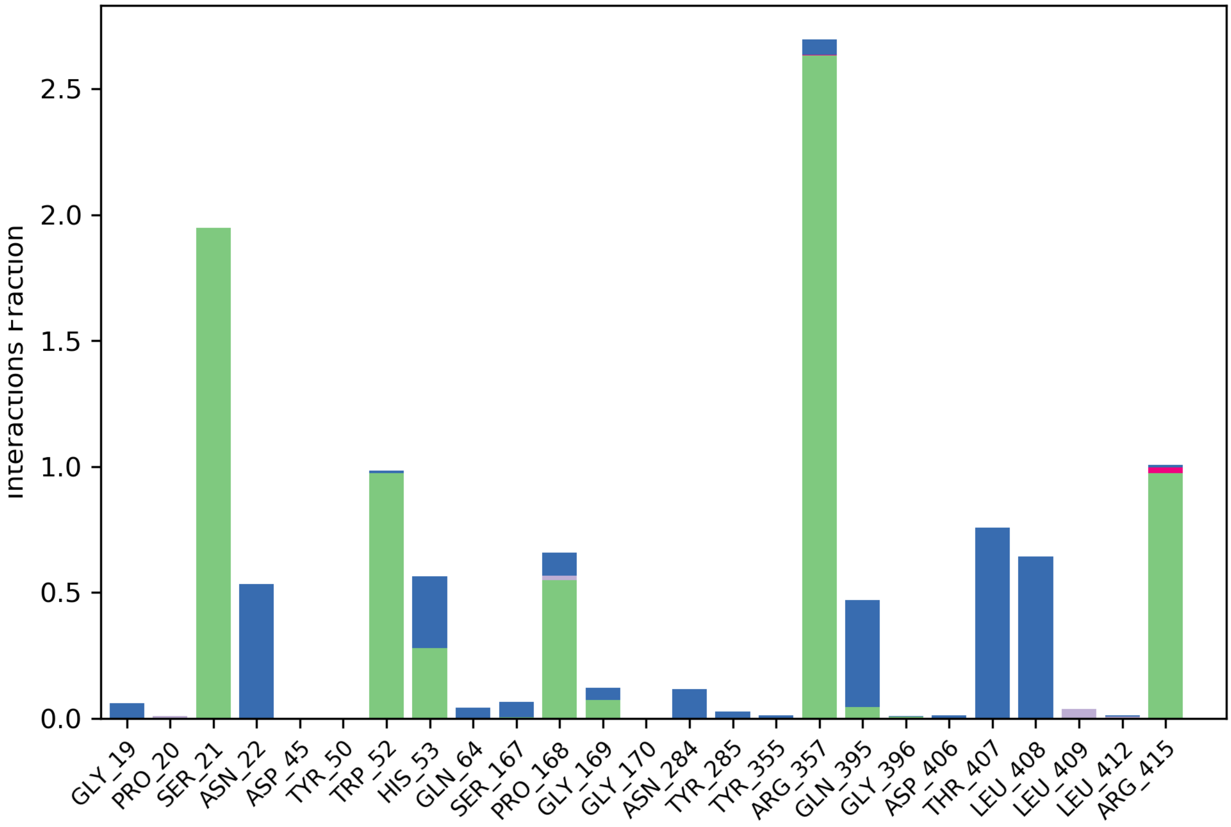

Figure S7: Various interactions of the ligand with the amino acids are represented. Amino acids of PvdA that make interactions with ligand and the type of interaction are provided. Green- Hydrogen Bond; Red-Ionic; Lavender-Hydrophobic; Blue-Water Bridge. Arg357 is found to make three kinds of interactions, namely, a hydrogen bond, water-bridge and ionic interaction.

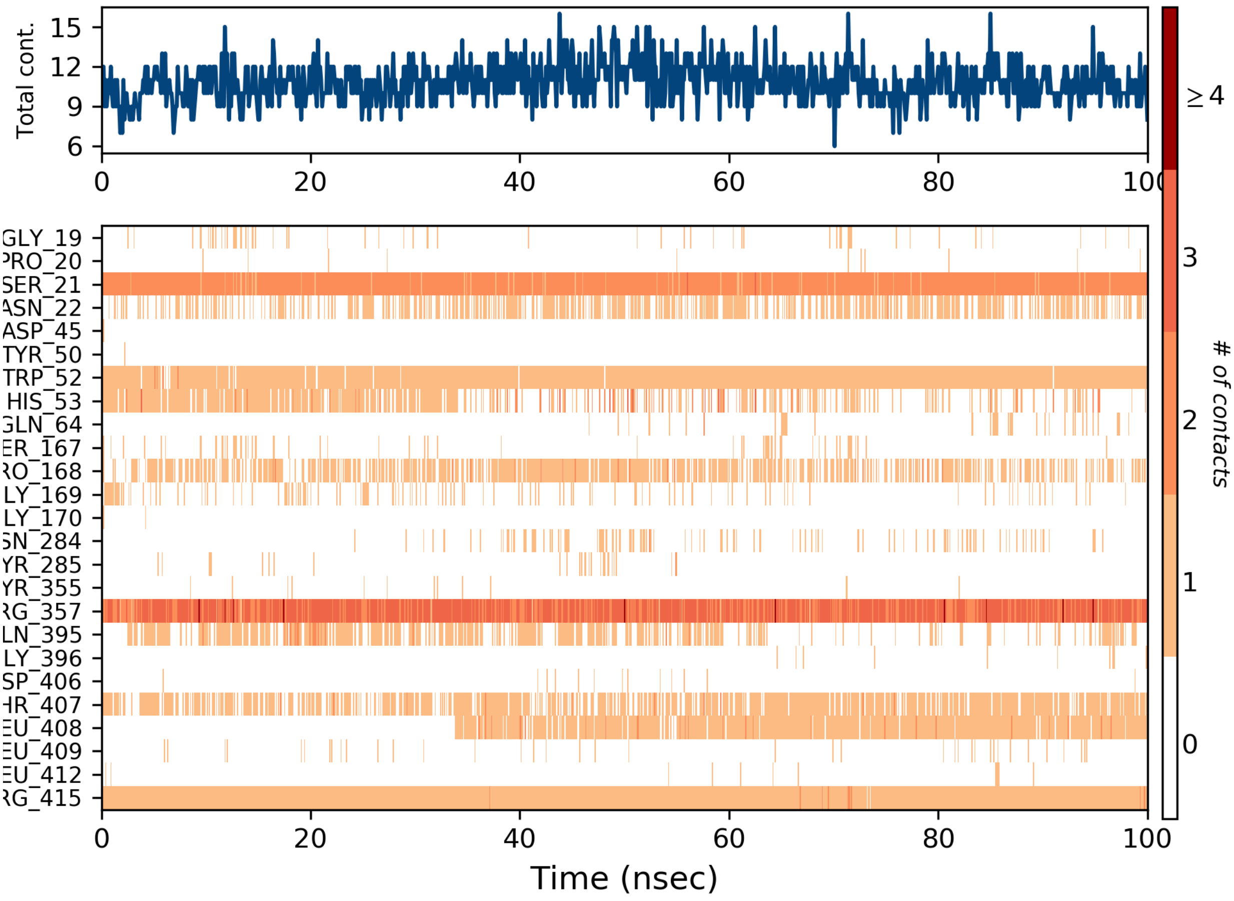

Figure S8: The number of contacts made by the ligand throughout the 100 ns simulation is shown in the top panel. Some residues make more than one specific contact with the ligand, which is represented by a darker shade of orange in the bottom panel, according to the scale to the right of the plot. Arg357 was found to make more than one single contact with the ligand.
